# MXene‐Coated, Multi‐Layered Mulberry Paper‐Based Flexible Tactile Sensor With High Sensitivity Over a Wide Pressure Range

**DOI:** 10.1002/smll.74205

**Published:** 2026-06-15

**Authors:** Sangrim Lee, Chaemin Won, Jaebeen Ahn, Wonkeun Park, Eunsoo Ha, Jongbaeg Kim, Taewook Kim, Changyong Yim, Yunsung Kang

**Affiliations:** ^1^ School of Semiconductor Convergence Engineering Kyungpook National University Daegu Republic of Korea; ^2^ Department of Precision Mechanical Engineering Kyungpook National University Sangju Gyeongbuk Republic of Korea; ^3^ School of Advanced Science and Technology Convergence Kyungpook National University Sangju Gyeongbuk Republic of Korea; ^4^ Department of Energy Chemical Engineering Kyungpook National University Sangju Gyeongbuk Republic of Korea; ^5^ School of Mechanical Engineering Yonsei University Seoul Republic of Korea; ^6^ Convergence Research Center of Mechanical and Chemical Engineering (CRCMCE) Kyungpook National University Sangju Gyeongbuk Republic of Korea

**Keywords:** flexible, mulberry paper, MXene, tactile sensors, wide pressure range

## Abstract

Highly sensitive, paper‐based tactile sensors utilizing conductive nanomaterials have attracted significant attention due to their porosity, foldability, and mechanical flexibility. However, achieving sensitivity above 10 kPa^−1^ across a wide pressure range remains a key challenge. In this study, we present a flexible tactile sensor based on stacked mulberry paper coated with Ti_3_C_2_T_x_ MXene. Owing to the hydrophilic nature of mulberry paper, the MXene layers conformally coat the fibrous network via dip coating. The rough, porous surface and multi‐layered architecture enhance contact resistance modulation, enabling high sensitivity (>15 kPa^−1^) over a broad pressure range (1–1000 kPa). We systematically investigate the effects of paper type, stacking configuration, and MXene loading to optimize sensor performance. The resulting device exhibits rapid response, durability over 1000 loading cycles, and consistent reproducibility. Its high flexibility and paper–fabric‐based structure allow seamless integration into wearable platforms such as gloves and wristbands, enabling real‐time monitoring of finger motion, arterial pulse, and touch intensity. Additionally, the wide detection range supports applications in Morse code signaling and CPR training feedback systems through wireless communication. These findings highlight MXene‐coated mulberry paper as a scalable, durable, and cost‐effective platform for wearable electronics requiring a balanced combination of high sensitivity and broad‐range pressure detection.

## Graphical abstract

Multi‐layered mulberry paper coated with MXene is used as a pressure‐sensing element in a resistive tactile sensor. The sensor achieves a high sensitivity exceeding 15 kPa^−1^ over a wide pressure range of 1–1000 kPa, demonstrating a balanced combination of sensitivity and sensing range among paper‐based tactile sensors incorporating MXene. In addition, the wide detection range with high sensitivity enables a proof‐of‐concept CPR training system.

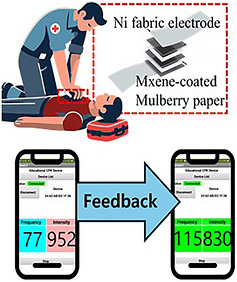

## 1 Introduction

Paper has attracted significant interest as a substrate for flexible electronics due to its low cost, mechanical flexibility, recyclability, ease of large‐scale production, and environmental sustainability [1, 2]. Additionally, paper exhibits porosity and a fibrous, rough surface, which enhance its performance as a pressure‐sensing medium in resistive tactile sensors by promoting greater changes in contact area under pressure [3, 4]. Consequently, paper has been employed as a structural component in tactile sensors incorporating conductive nanomaterials such as carbon nanotubes [5], graphene [6], transition metal dichalcogenides [7], and metal nanowires [8] to enable effective modulation of contact resistance. Among these conductive materials, two‐dimensional transition metal carbides and nitrides (MXenes) have recently gained considerable attention owing to their high electrical conductivity and abundance of surface functional groups [9]. Their layered lamellar structure, held together by van der Waals forces and hydrogen bonding, allows for dynamic interlayer spacing under applied stress, making them well suited for piezoresistive sensing applications [10, 11]. Furthermore, MXenes demonstrate strong resistance to environmental degradation, enhancing their reliability in practical applications [12]. In parallel, significant progress has recently been made in cellulose‐ and natural fiber‐based flexible sensors, where porous fibrous architectures are used as functional structural components for humidity, pressure, and multimodal tactile sensing [13, 14]. Recent studies have demonstrated MXene‐functionalized paper‐based humidity sensors, plant fiber composite‐based capacitive pressure sensors, and self‐powered tactile sensors capable of pressure and temperature detection, highlighting the broad utility of natural fibrous substrates in flexible electronics [15]. In addition, MXenes have been widely adopted as conductive materials in flexible tactile sensors integrated with various paper substrates, including cellulose, tissue, filter, and printing paper [10, 11, 16, 17, 18, 19, 20, 21, 22, 23, 24, 25]. For example, Yang et al. reported a MXene‐based tactile sensor on tissue paper that exhibited ultrahigh sensitivity of 509.5 kPa^−1^ in the pressure range of 0.5–10 kPa [19, 26, 27, 28]. Building upon these developments, significant progress has recently been made in flexible tactile and self‐powered sensors, where porous, fibrous, microstructured, or composite architectures are used to improve sensitivity, breathability, mechanical robustness, and multimodal sensing [26, 27, 28, 29]. However, many MXene‐based, paper‐structured tactile sensors reported to date exhibit reduced sensitivity at higher pressures (hundreds of kPa), which restricts their usability in applications requiring high sensitivity across a broader pressure range, such as gait monitoring or force detection during exercise [30]. One approach to achieving high sensitivity across a broad pressure range is the use of a multilayer structure, which promotes even stress distribution across the layered arrangement under applied pressure, thereby increasing the contact area among the layers [31, 32]. This enables the simultaneous realization of high sensitivity and a wide pressure range. For example, Pyo et al. reported a high sensitivity of 26.13 kPa^−1^ at pressures up to 982 kPa using a multilayered fabric structure [32].

Meanwhile, mulberry paper, also known as Korean paper (Hanji), has attracted attention as a novel alternative to conventional paper substrates due to its high mechanical strength and chemical resistance [33]. The high proportion of holocellulose in mulberry paper contributes to its enhanced mechanical strength and chemical durability [34], while providing abundant –OH groups that promote its hydrophilic nature [35]. This hydrophilicity facilitates solution‐based processing, making fabrication more scalable and straightforward. Additionally, the fibers in mulberry paper are longer and thicker than those in other types of paper, further improving its strength and porosity [33]. Thus, these characteristics make mulberry paper an excellent candidate as a substrate material for flexible tactile sensors. For instance, Lee et al. developed stacked mulberry paper‐based tactile sensors that exhibited high sensitivity exceeding 1 kPa^−1^ over a wide pressure range of 0.05–900 kPa [36]. Inkjet printing has been employed for precise patterning and cost‐effective deposition of pressure‐sensing materials such as carbon nanotubes (CNTs) [36, 37]. However, inkjet‐printed CNT networks inherently form localized, planar conductive junctions that limit the number of effective electronic carrier pathways and often lead to saturation in contact‐resistance modulation under high pressure. As previous studies have shown, the electrical conductivity of sensing materials is a critical factor in enhancing pressure sensitivity in resistive tactile sensors [38]. Guided by this design principle, we adopted a dip‐coating process to conformally deposit Ti_3_C_2_T_x_ MXene onto the three‐dimensional fibrous network of mulberry paper, enabling the formation of dense, continuous conductive pathways along fiber–fiber and layer–layer interfaces and resulting in a pronounced evolution of contact resistance under applied pressure. Additionally, the influence of different types of mulberry paper on the performance of tactile sensors has not yet been systematically investigated.

## 2 Results and Discussion

**FIGURE 1:**
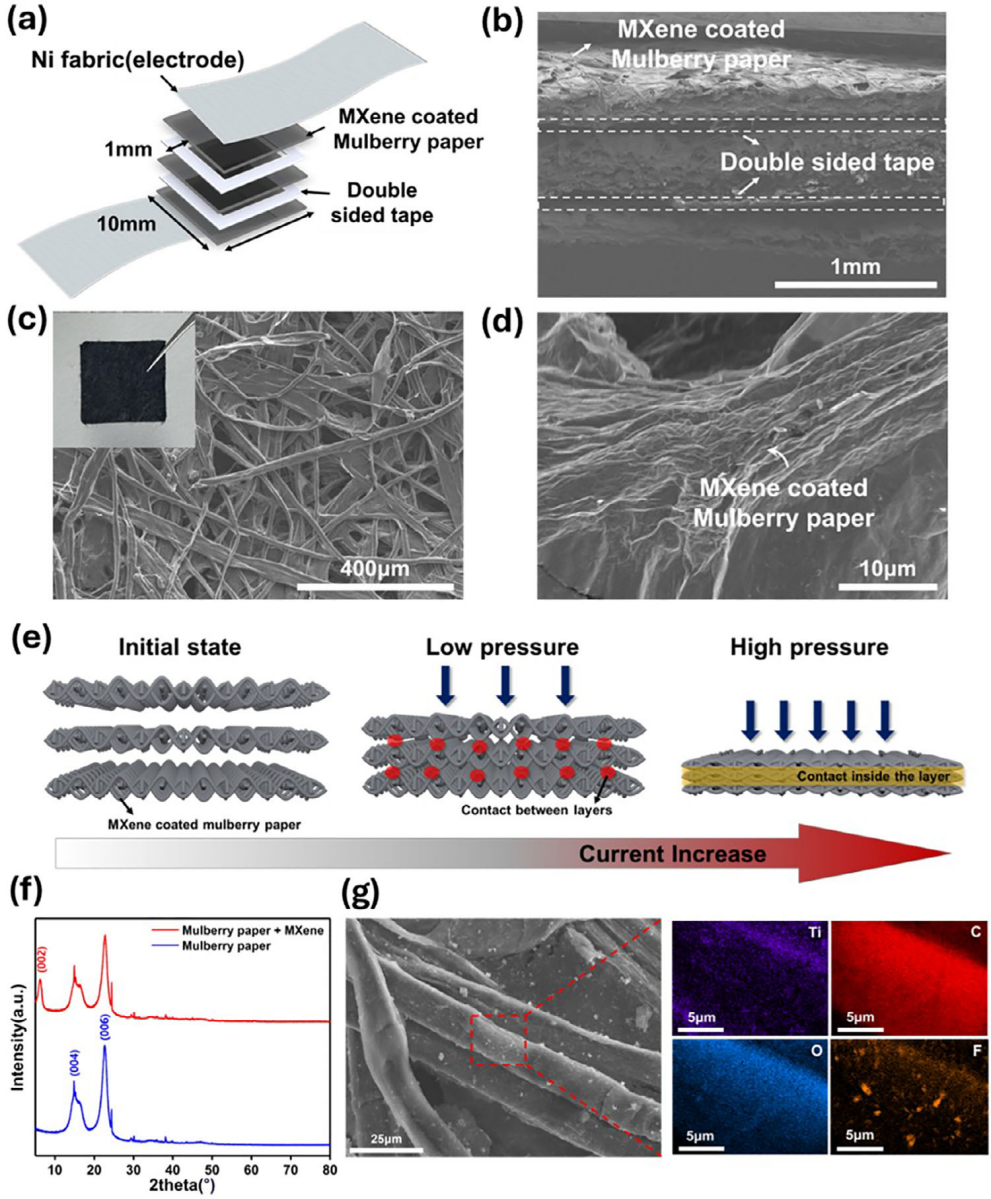
(a) Schematic illustration of the proposed pressure sensor integrated with Ni‐coated fabric‐based electrodes. (b) Cross‐sectional SEM image of the stacked sensor assembly. (c) Top‐view SEM image of MXene‐coated mulberry paper (inset: optical image of the coated paper). (d) High‐magnification SEM image showing the detailed morphology of MXene layers on the fiber surface. (e) Schematic of the pressure detection mechanism based on changes in contact resistance under applied pressure. At relatively low pressure, electrical contact points are primarily formed between adjacent layers. As pressure increases, additional pathways are established through inter‐fiber contacts within the porous structure of the mulberry paper, resulting in increased conductivity. (f) XRD spectra of pristine and MXene‐coated mulberry paper. A dominant (002) peak characteristic of Ti_3_C_2_T_x_ MXene appears at a 2θ angle of ∼6.2°. Additional peaks at ∼15.0° and 22.6°, observed in both spectra, originate from the mulberry fibers. (g) SEM image and corresponding EDX elemental mapping of MXene‐coated mulberry paper. Uniform distribution of Ti confirms conformal coating of Ti_3_C_2_T_x_ MXene on the fibrous network.

Figure S1a illustrates the preparation protocol of Ti_3_C_2_T_x_ MXene via the minimally intensive layer delamination (MILD) method (see Experimental Section for details) [39]. A few‐layered Ti_3_C_2_T_x_ MXene was successfully obtained and suspended in deionized (DI) water. Figure S1b shows the fabrication process of the proposed sensor. Initially, mulberry paper was dip‐coated with the Ti_3_C_2_T_x_ solution and subsequently dried in a vacuum oven. The coated paper was then cut into 10 × 10 mm^2^ pieces, and three such pieces were carefully stacked and adhered using double‐sided tape. Figure 1a presents a schematic illustration of the proposed sensor, which includes electrodes composed of nickel (Ni) fabric. Commercially available Ni‐coated fabrics were selected as electrodes due to their compatibility with wearable applications, as discussed later. Figure 1b shows a cross‐sectional view of the stacked MXene‐coated mulberry paper, highlighting its long and thick fibers. This fiber structure is further confirmed by the top view in the center of Figure 1c, which also reveals the porous and rough surface of the coated paper, an advantageous feature for maximizing contact area changes under varying pressures. The inset of Figure 1c displays an optical image of the MXene‐coated mulberry paper. Similarly, Figure 1d provides another SEM image that showcases the microscale surface roughness, further illustrating the structural advantages of the MXene‐coated mulberry paper. Figure 1e conceptually illustrates the pressure‐sensing mechanism, highlighting the role of the MXene coating in modulating contact resistance. We employed a multi‐layered structure of MXene‐coated mulberry paper, which increases the contact area and effectively distributes stress across the layers [31, 32]. This architecture is highly advantageous for achieving high sensitivity over a wide pressure range compared to a single‐layer configuration. In the initial state, the hierarchical and porous network of the MXene‐coated fibers maintains minimal contact points, resulting in high contact resistance and low current flow. Upon applying pressure, the contact area between the conductive MXene layers on the fiber surfaces increases, both within individual layers and across interlayer interfaces. This formation of additional conductive pathways facilitates a significant increase in current flow. Sensitivity is further enhanced by the intrinsic microscale roughness and flexibility of the mulberry paper, which allows for continuous and effective MXene‐to‐MXene contact modulation over a wide pressure range. In other words, as the number of layers increases, the interfacial contact area between MXene‐coated layers also increases, resulting in a greater current change under the same applied pressure and thereby enhancing sensitivity. The role of Ti_3_C_2_T_x_ MXene in this architecture is not limited to providing electrical conductivity. Recent MXene‐based flexible sensors have shown that MXene can enhance sensing performance by forming conductive networks, regulating interfacial contact resistance, and improving pressure‐sensitive charge transport in porous or composite structures [40, 41, 42]. In the present sensor, the high electrical conductivity of Ti_3_C_2_T_x_ MXene enables efficient charge transport along the mulberry paper fibers, while its hydrophilic surface chemistry and abundant terminal functional groups facilitate conformal coating on the hydrophilic fibrous network. The two‐dimensional layered morphology of MXene further provides pressure‐sensitive contact interfaces at fiber–fiber and layer–layer junctions. Therefore, the MXene‐coated mulberry paper structure enhances contact‐resistance modulation under compression, contributing to the high sensitivity, broad pressure detection range, and reproducible sensing behavior of the proposed sensor. Figure 1f shows the XRD patterns of pristine mulberry paper and mulberry paper coated with Ti_3_C_2_T_x_ MXene. Compared with pristine mulberry paper, the MXene‐coated sample exhibits a pronounced diffraction peak at 2θ ≈ 6.2°, which is assigned to the (002) reflection of Ti_3_C_2_T_x_. This shift of the (002) peak to lower angles, relative to the characteristic (002) peak of the Ti_3_AlC_2_ MAX phase at approximately 10°, indicates an expanded interlayer spacing resulting from the successful delamination of Ti_3_C_2_T_x_ sheets after MILD etching, consistent with previous reports [43, 44]. In addition, the absence of the Ti_3_AlC_2_‐related diffraction peak at around 39°, which originates from the Al‐containing MAX phase, further confirms the effective removal of the Al layer and the formation of Ti_3_C_2_T_x_. Notably, the XRD measurement was performed on a thin MXene coating deposited on a fibrous mulberry paper substrate rather than on bulk or freestanding MXene films. Under such conditions, higher‐order MXene reflections are often weak or suppressed due to the low MXene loading, preferred orientation of restacked sheets, and the strong background contribution from the cellulose‐based substrate. Therefore, the presence of a dominant (002) peak at ∼6.2° is considered a reliable structural signature of successfully delaminated and restacked Ti_3_C_2_T_x_ sheets in coated or composite systems [43, 44]. Other peaks were observed at 2θ angles of approximately 15.0° and 22.6°, independent of the presence of MXene, which originate from the intrinsic crystalline structure of mulberry fibers [45]. To confirm the presence of the MXene coating on the mulberry fibers, we conducted EDX elemental mapping, which predominantly revealed Ti, C, O, and F, as illustrated in Figure 1g. The detected fluorine originates from intrinsic –F surface terminations of Ti_3_C_2_T_x_ MXene produced via the MILD process, rather than from residual HF, which is not used in the synthesis [46, 47, 48, 49]. The uniform distribution of Ti confirms the conformal coating of Ti_3_C_2_T_x_ MXene on the mulberry paper fibers. To verify that the Ti signal originates solely from the MXene, we also performed EDX elemental mapping on pristine mulberry paper (Figure S2), where Ti was not detected, confirming that the base material consists primarily of carbon and oxygen.

**FIGURE 2:**
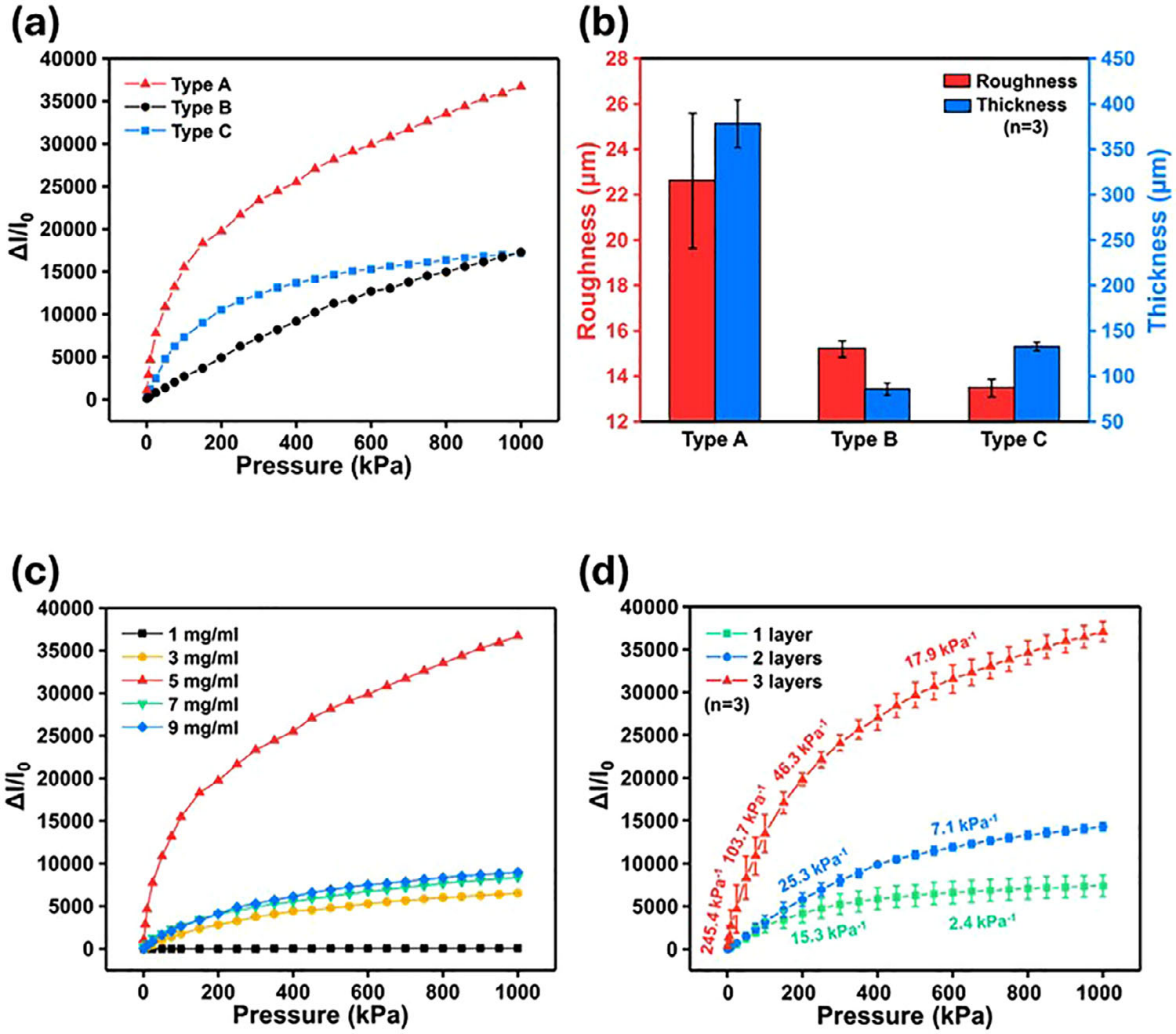
(a) Relative current change of three mulberry paper‐based tactile sensors fabricated using different paper types. Type A sensors exhibit a greater relative current response to increasing pressure compared to Type B and Type C sensors. (b) Surface roughness and thickness measurements of Type A, B, and C mulberry paper. The enhanced sensitivity of Type A sensors across a broad pressure range is attributed to their higher roughness and thickness. (c) Relative current response under applied pressure for sensors coated with varying concentrations of MXene. Sensors dip‐coated with 5 mg/mL MXene demonstrate notable sensitivity across a wide pressure range, attributed to reaching the percolation threshold. (d) Relative current change under applied pressure as a function of the number of sensing layers. The three‐layer configuration exhibits the highest sensitivity across contiguous pressure ranges of 1–10, 10–150, 150–350, and 350–1000 kPa, respectively. Each data point represents the mean value, with error bars indicating the standard deviation (*n* = 3). The different sensitivity slopes correspond to the sequential mechanical deformation stages of the multi‐layered fibrous structure.

We investigated the effects of differently prepared mulberry paper substrates on pressure detection. Three types of mulberry paper were denoted as Type A, Type B, and Type C. Because the detailed manufacturing conditions of the commercial papers were not fully disclosed by the supplier, we did not assign the observed differences among the substrates to specific manufacturing steps. Instead, the comparison was based on experimentally measurable substrate parameters, including fiber thickness, surface roughness, basis weight, apparent density, and SEM‐based fiber‐network morphology, as summarized in Table S1 and Figure S3. A previous study on mulberry paper has shown that its structural and mechanical properties can vary depending on manufacturing‐related variables, such as sheet‐forming method, fiber orientation, lamination, and post‐treatment [50]. Therefore, the observed differences in the measured structural parameters can reasonably affect the pressure‐sensing behavior of mulberry paper‐based tactile sensors. The relative change in current (Δ*I*/*I*_0_) was evaluated under a bias voltage of 1 V (see Figure S4 and Experimental Section for the pressure‐sensing setup). As shown in Figure 2a, the Type A‐based tactile sensor exhibits a greater change in relative current with increasing pressure compared to those of Type B‐ and Type C‐based sensors. To investigate this behavior, we compared the measured structural parameters of the three substrates, as shown in Figure 2b and Table S1. Among the three substrates, Type A exhibited the largest fiber thickness and surface roughness. The thicker fibers of Type A are likely associated with a more pronounced fibrous network and larger surface undulations, which can increase the measured surface roughness. In the relatively low‐pressure range, this rough fibrous surface promotes interlayer contact‐area modulation between MXene‐coated paper layers, resulting in a larger change in contact resistance. At higher pressures, the porous and structurally thicker fibrous network of Type A provides a larger deformable volume for internal fiber‐network compression and contact‐resistance variation. Therefore, the superior pressure‐sensing performance of the Type A‐based sensor is attributed to the combined effects of thicker fibers, higher surface roughness, and a more deformable porous fibrous structure. Based on these results, the characterization presented hereafter is based on the Type A sensor.

Another factor influencing the change in contact resistance is the amount of MXene deposited on the mulberry paper fibers. We examined the effect of MXene loading on pressure sensing performance by varying the solution concentration for dip‐coating to 1, 3, 5, 7, and 9 mg/mL. The relative current change under applied pressure for sensors coated with different MXene concentrations is shown in Figure 2c. A three‐layer stacked structure was consistently applied to all sensors in this experiment. The superior response at 5 mg/mL can be attributed to the formation of an optimized conductive network near an effective percolation regime [51]. At low MXene loading, the amount of conductive material deposited on the mulberry fibers is insufficient to form continuous conductive pathways, resulting in high initial resistance and limited pressure‐induced current modulation. At an intermediate loading, pressure‐induced fiber–fiber and layer–layer contacts can effectively modulate the conductive pathways, leading to the largest contact‐resistance modulation and sensitivity. In contrast, excessive MXene loading forms highly connected conductive pathways even before pressure is applied, which lowers the initial resistance but reduces the relative contribution of additional pressure‐induced contact formation. Consequently, the relative current change, Δ*I*/*I*_0_, and sensitivity decrease or become saturated. This behavior is consistent with the general percolation‐based sensing mechanism reported for resistive tactile sensors using conductive nanomaterials such as CNTs, graphene, and MXene, where sensitivity is often maximized near the percolation threshold because the conductive network is highly sensitive to pressure‐ or strain‐induced changes in contact resistance [52, 53, 54]. Similar concentration‐dependent behavior has also been reported in MXene‐based pressure sensors, where optimized MXene loading or coating concentration maximized the sensing response by balancing conductive network formation and pressure‐sensitive resistance modulation [55, 56]. To eliminate the possibility that the percolation threshold was not reached in B‐ and C‐type mulberry paper‐based sensors, we also compared the effect of MXene loading on pressure sensing performance in these substrates, as shown in Figure S5. These results confirm that A‐type mulberry paper, with its high surface roughness and greater thickness, is highly advantageous for achieving high sensitivity across a wide pressure range. Additionally, to verify the advantage of using mulberry paper as a structural material, we compared the relative change in current of sensors composed of Kent and oil paper with the same configuration (Figure S6). The relative current change in these sensors was significantly lower than that of the mulberry paper‐based sensor, implying that the use of mulberry paper is highly favorable for resistive tactile sensors. Figure 2d shows the relative current change under pressure as a function of the number of MXene‐coated mulberry paper sensing layers. The measurements were repeated using three independently fabricated sensors for each layer configuration, and the data are presented as the mean value and standard deviation (*n* = 3). Compared with the one‐ and two‐layer devices, the three‐layer sensor exhibited a larger relative current change over the entire pressure range, which is attributed to the increased contact area and more effective stress distribution between sensing layers. To provide a continuous sensitivity analysis over the main working range, the pressure response of the three‐layer sensor was fitted using four contiguous pressure regions of 1–10, 10–150, 150–350, and 350–1000 kPa. The corresponding sensitivities were 245.4, 103.7, 46.3, and 17.9 kPa^−1^, respectively. This enhancement originates from the increased contact area and efficient stress distribution between sensing layers, which are further supported by the cross‐sectional SEM observations under compression (Figure S7) [31, 32]. To further support the structural role of the multilayered mulberry paper under compression, cross‐sectional SEM images were obtained for the three‐layered MXene‐coated mulberry paper sensor under different compression states (Figure S7). Because the SEM compression stage does not provide calibrated pressure values, the images are presented as qualitative observations under uncompressed, moderately compressed, and highly compressed states. In the uncompressed state, the stacked mulberry paper layers retain interlayer gaps and a porous fibrous network. Under moderate compression, the interlayer spacing decreases, and layer–layer contact becomes more pronounced, increasing the number of conductive pathways between adjacent MXene‐coated layers. Under higher compression, the internal fibrous network within each layer becomes more compact, and intralayer fiber–fiber contact becomes more pronounced. This sequential deformation from interlayer contact formation to intralayer fiber–fiber densification supports the sustained contact‐resistance modulation over a broad pressure range. Additionally, the effect of increasing the number of layers is analogous to increasing the overall thickness, as discussed in Figure 2d. However, when the number of layers exceeds three (i.e., four or five layers), the extracted sensitivity decreases, as shown in Figure S8. This trend primarily originates from the inherently high and unstable initial resistance (>MΩ) of high‐layer‐count devices [32, 36], which necessitates a conservative definition of the initial current (*I*_0_) to avoid sensitivity overestimation caused by electrical noise. As a result, the calculated sensitivity for four‐ and five‐layer devices appears lower when evaluated under identical and conservative criteria. In addition, excessive stacking may increase the overall stiffness of the sensing structure, which could further limit effective deformation and constrain the pressure‐induced evolution of the internal conductive network. Based on the reliability and comparability of the sensitivity evaluation, we selected the three‐layered structure as the standard configuration and employed it consistently throughout this study. In the proposed sensor architecture, pressure‐sensing performance is governed by the coupled effects of MXene loading, paper structural properties, and pressure‐induced deformation, all of which modulate the evolution of contact resistance under applied pressure. The amount of MXene coated on the mulberry paper fibers determines the formation of conductive pathways, where an optimal loading near the percolation threshold enables significant resistance modulation in response to mechanical deformation. Meanwhile, the thickness and surface roughness of the mulberry paper influence the effective contact area and deformability of the fibrous network, thereby affecting the number and stability of pressure‐activated contact junctions. Upon pressure application, local deformation of the multilayered fibrous structure induces dynamic rearrangement of MXene‐coated contacts, leading to pronounced changes in contact resistance. These factors collectively contribute to the high sensitivity and wide pressure detection range of the sensor, while maintaining stable and reproducible signal characteristics when operated within the optimized design window defined in this study.

**FIGURE 3:**
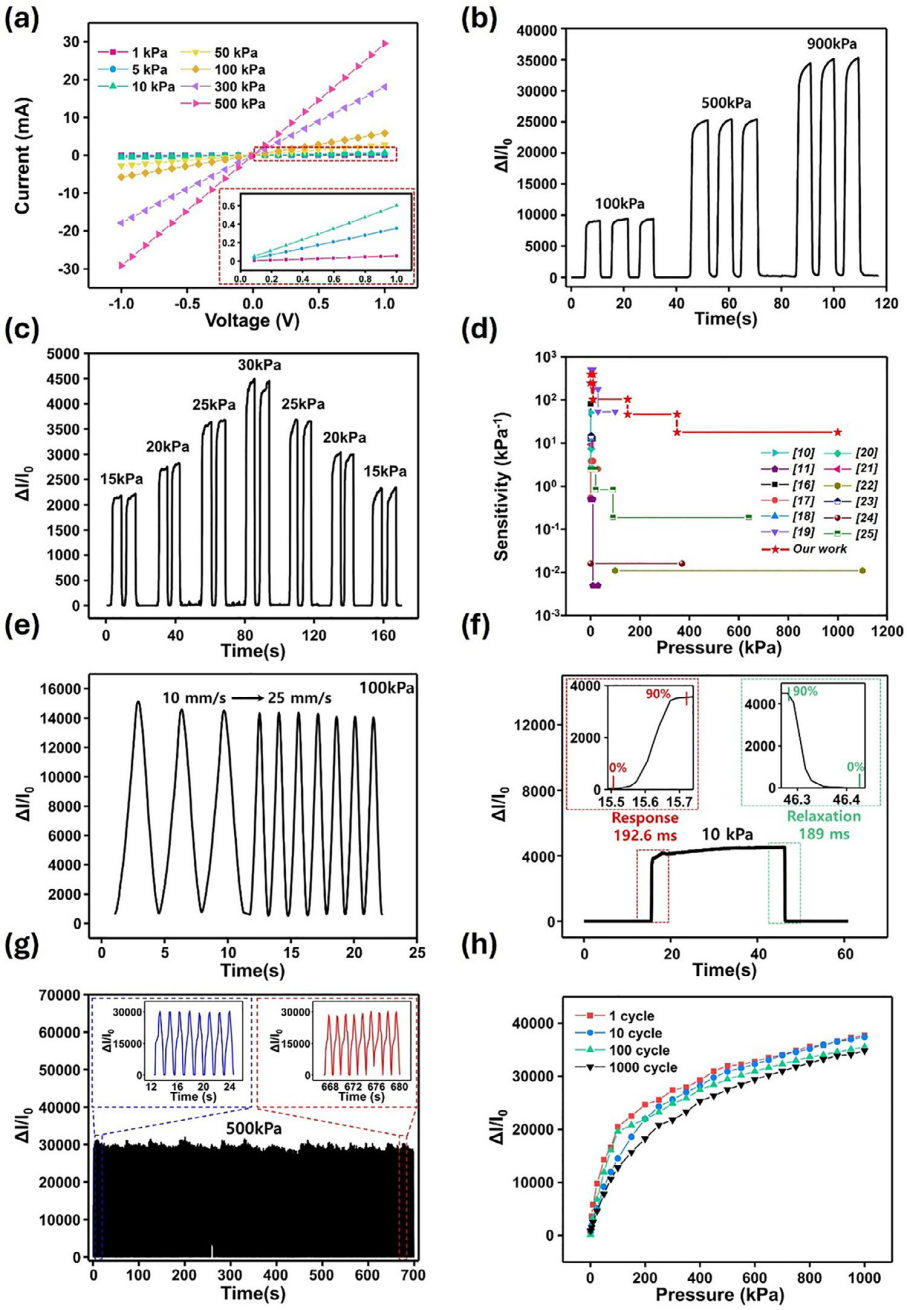
(a) Current–voltage (*I–V*) characteristics under varying pressure conditions. As the applied pressure increased from 1 to 500 kPa, the resistance, derived from the linear curves, significantly decreased from 17.4 kΩ to 34.33 Ω. (b, c) Dynamic response of the sensor through loading and unloading cycles within a range of (b) 100 to 900 kPa and (c) 15 to 30 kPa demonstrates consistent and rapid reaction to repeated application and release of pressure. (d) Comparison of the sensing performance of our sensor with that of paper substrate‐based tactile sensors with MXene shows that the proposed sensor maintained high sensitivity, exceeding 15 kPa^−1^ over a wide pressure range up to 1000 kPa. (e) Dynamic sensing performance under varying pressing speeds from 10 to 25 mm/s verified the fast response of our sensor. (f) The response and recovery times were measured as 192.6 and 189 ms, respectively, defined as the time to reach 90% of the steady‐state signal from the initial baseline (0%) and vice versa. (g) Durability of the sensor under repeated high‐pressure loading of 500 kPa indicates high mechanical stability of the MXene and mulberry paper‐based design. (h) Relative change in current over pressure after 1, 10, 100, and 1000 loading cycles highlights the sensor's stability under repeated pressure cycles.

The pressure‐sensing performance of the tactile sensor based on MXene‐coated mulberry paper was evaluated under various loading conditions. Figure 3a presents the current–voltage (*I–V*) characteristics of the sensor under different applied pressures. As the pressure increased from 1 to 500 kPa, the sensor exhibited a significant decrease in resistance, from 17.4 kΩ to 34.33 Ω, as determined from the linear *I–V* curves. The dynamic response of the sensor was further assessed through cyclic loading and unloading tests within pressure ranges of 100–900 and 15–30 kPa, as shown in Figure 3b,c, respectively. The sensor demonstrated a stable and rapid response to repeated pressure application and release, indicating excellent dynamic performance. To further investigate the limit of detection (LOD), we evaluated the dynamic response of the sensor under low pressures of 98, 490, and 981 Pa using calibrated load weights, as shown in Figure S9a. Clear and reproducible current responses were observed, demonstrating reliable pressure discrimination well below 1 kPa. The LOD was defined as the minimum pressure that generates a signal exceeding three times the root‐mean‐square noise level of the baseline current. To validate this criterion, we applied an extremely small pressure of approximately 1.96 Pa using a lightweight object (a grain of rice), as shown in Figure S9b. Despite the presence of minor electrical noise, the measured signal was distinctly greater than baseline fluctuations, confirming that the sensor can detect pressures on the order of a few pascals. These results demonstrate that the sensor maintains high sensitivity across both low‐ and high‐pressure regimes. Figure 3d compares the sensing performance of our sensor with previously reported paper substrate‐based tactile sensors incorporating MXene (see Table S2 for detailed values) [10, 11, 16, 17, 18, 19, 20, 21, 22, 23, 24, 25]. Our sensor exhibited a comparable sensitivity of 245.4 kPa^−1^ in the low‐pressure range of 1–10 kPa, approaching the performance of a previously reported sensor with 509.5 kPa^−1^ sensitivity in the 0.5–10 kPa range [19]. Notably, while several previously reported MXene‐based paper tactile sensors have demonstrated broad pressure detection ranges, their sensitivity generally decreases in the high‐pressure regime. In contrast, the proposed sensor maintains sensitivity above 15 kPa^−1^ over a broad pressure range up to 1000 kPa, indicating that high sensitivity and wide‐range pressure detection can be achieved simultaneously in the present MXene‐coated mulberry paper architecture. The primary distinction of this study, compared to previous MXene‐based tactile sensors, lies in the use of mulberry paper as an active structural substrate rather than a passive support. While earlier studies have employed various paper substrates, including cellulose paper [16, 18, 20, 21, 23, 24, 57], printing paper [22], tissue [17, 19], filter paper [11, 25], and airlaid paper [58], these materials typically consist of relatively short and thin fibers and primarily function as mechanical supports for the conductive layer. In contrast, mulberry paper comprises long and thick fibers that provide both high mechanical strength and pronounced porosity [33]. Under low applied pressure, the highly porous fibrous architecture of mulberry paper facilitates the formation of numerous initial point contacts between adjacent MXene‐coated fibers and layers, resulting in a rapid increase in effective contact area and high sensitivity. Dip coating enables conformal coverage of curved fiber surfaces and inter‐fiber junctions with MXene, forming a dense three‐dimensional conductive network that offers significantly more conductive pathways than planar patterning methods such as inkjet printing [36]. As pressure increases, progressive activation of interlayer contacts within the multilayer structure drives a continuous evolution of contact resistance, thereby sustaining sensitivity over an extended pressure range. At higher pressures, the intrinsic mechanical robustness of mulberry paper prevents structural collapse and redistributes the applied stress across layers, effectively delaying signal saturation. Through the synergistic integration of the unique fibrous structure of mulberry paper, the conductively dense MXene network formed via dip coating, and the multilayer architecture that promotes gradual contact evolution and uniform stress distribution, the proposed sensor achieves high sensitivity across a wide pressure range, clearly distinguishing it from previously reported MXene–paper‐based tactile sensors. This highlights that our approach, utilizing a stacked structure of mulberry paper coated with MXene, is highly advantageous for achieving high sensitivity across a wide pressure range. Furthermore, we compared the performance of our sensor with that of recently reported MXene‐based tactile sensors, as shown in Figure S10 [59, 60, 61, 62, 63, 64, 65, 66]. Although several previous studies have demonstrated pressure detection ranges extending into the megapascal range [60, 61, 63], their sensitivities are relatively limited in the lower‐pressure regime. In contrast, the proposed sensor exhibits significantly higher sensitivity within the pressure range up to 1000 kPa. Figure 3e presents the dynamic sensing performance under varying pressing speeds from 10 to 25 mm/s, confirming the fast response of our sensor. The response and recovery times were measured as 192.6 and 189 ms, respectively, as shown in Figure 3f. As illustrated in Figure 3f, these temporal characteristics were defined as the time interval required for the current signal to reach 90% of its steady‐state value from the initial baseline (0%) (and vice versa for recovery) [67, 68]. Since both times remained below 250 ms even under a pressure of 10 kPa, the sensor is capable of detecting dynamic pressures up to tens of kPa at frequencies of up to 4 Hz. To examine whether these temporal characteristics are affected by the magnitude of applied pressure, we performed additional measurements at higher pressure levels of 250 and 500 kPa. As shown in Figure S11, the response/relaxation times were 315/342 ms at 250 kPa and 270/234 ms at 500 kPa, respectively, indicating that the sensor maintained sub‐second dynamic response over the tested pressure range.

As illustrated in Figure 3g, the sensor exhibited high durability under repeated high‐pressure loading of 500 kPa. Furthermore, the Δ*I*/*I*_0–_ *pressure* curves in Figure 3h remained largely unchanged after 1000 loading cycles, demonstrating the sensor's stability under repeated pressure application. A slight downward shift in the baseline response observed after repeated loading is attributed to an initial mechanical conditioning process, during which the fibrous network of the mulberry paper and the interfacial contacts between MXene‐coated fibers undergo gradual rearrangement and densification. This leads to a more stable contact configuration rather than irreversible degradation. To further verify long‐term durability, the cyclic loading test was extended to 2500 loading–unloading cycles at a pressure of 500 kPa, as shown in Figure S12. The sensor maintained stable and repeatable pressure responses throughout the extended cycling without abrupt degradation or failure. This robust durability is primarily attributed to the intrinsically high mechanical strength of the mulberry paper substrate, which effectively withstands repeated compressive stress [34]. Durability under dynamic bending deformation was also evaluated. Figure S13 compares the relative current change before and after 1000 cycles of harsh bending at an angle of approximately 90°, representing a severe mechanical condition. Although a reduction in response is observed after bending, the sensors remain functional. This behavior is consistent with previous reports on two‐dimensional conductive materials, where large bending deformation may induce microcracks in the conductive layer while preserving sensing capability [69]. To simulate more realistic wearable conditions, a moderate bending test was conducted, consisting of 1000 cycles at a bending radius of 0.6 cm. As shown in Figure S14, the sensor maintains high sensitivity on the order of tens of kPa^−1^ over a pressure range up to 500 kPa, demonstrating sufficient functional durability to endure dynamic mechanical strains relevant to wearable applications. In addition to mechanical durability, environmental stability was assessed, as paper‐based devices are often exposed to varying ambient conditions during practical use. The pressure‐sensing performance was first evaluated across a temperature range from room temperature to 62.7°C, as shown in Figure S15. The sensor exhibited stable and repeatable responses with negligible variation, indicating sufficient thermal stability for typical wearable operating conditions. The effect of relative humidity (RH) was further investigated, as shown in Figure S16. Across a wide humidity range (50%–90% RH), the sensor maintained a large relative current change, reaching approximately Δ*I/I*_0_ ≈ 30 000 at 1000 kPa even under high‐humidity conditions. These results demonstrate that the proposed sensor preserves functional performance under temperature and humidity variations relevant to practical wearable applications. Furthermore, to verify functional reversibility and the need for recalibration, we measured the pressure‐dependent current response after 30 min exposure to elevated temperature (60°C), high humidity (80% RH), and combined temperature/humidity conditions (60°C and 80% RH). As shown in Figure S17, the Δ*I*/*I*_0–_pressure curves measured after exposure nearly overlapped with the initial curves after the sensors were returned to ambient conditions. These results indicate that the sensor exhibits largely reversible sensing behavior and does not require recalibration after short‐term exposure to the tested environmental conditions. Nevertheless, prolonged exposure to high humidity may still induce signal drift because Ti_3_C_2_T_x_ MXene can undergo oxidation in humid environments [70]; therefore, recalibration may be required for long‐term operation under highly humid conditions. To assess the reproducibility of the dip‐coated MXene–mulberry paper sensors, ten independent devices (*n* = 10) fabricated under identical conditions were evaluated, with their pressure‐sensing performances summarized in Figure S18. The sensors exhibited highly consistent pressure sensitivity and detection range, with the statistical analysis confirming minimal device‐to‐device variation. This robust reproducibility, derived from a larger sample size, underscores the reliability of our fabrication process. To further examine the spatial distribution of MXene on the mulberry paper substrate, EDX elemental mapping was conducted at three different positions on the dip‐coated samples, as shown in Figure S19. Although minor variations in the local atomic weight ratio were observed, the Ti element was consistently distributed across the fibrous network, confirming conformal MXene coating at the fiber scale. These results suggest that small‐scale variations in MXene coverage do not significantly affect overall sensor performance, owing to the contact‐based sensing mechanism that averages local variations across the three‐dimensional fibrous network.

**FIGURE 4:**
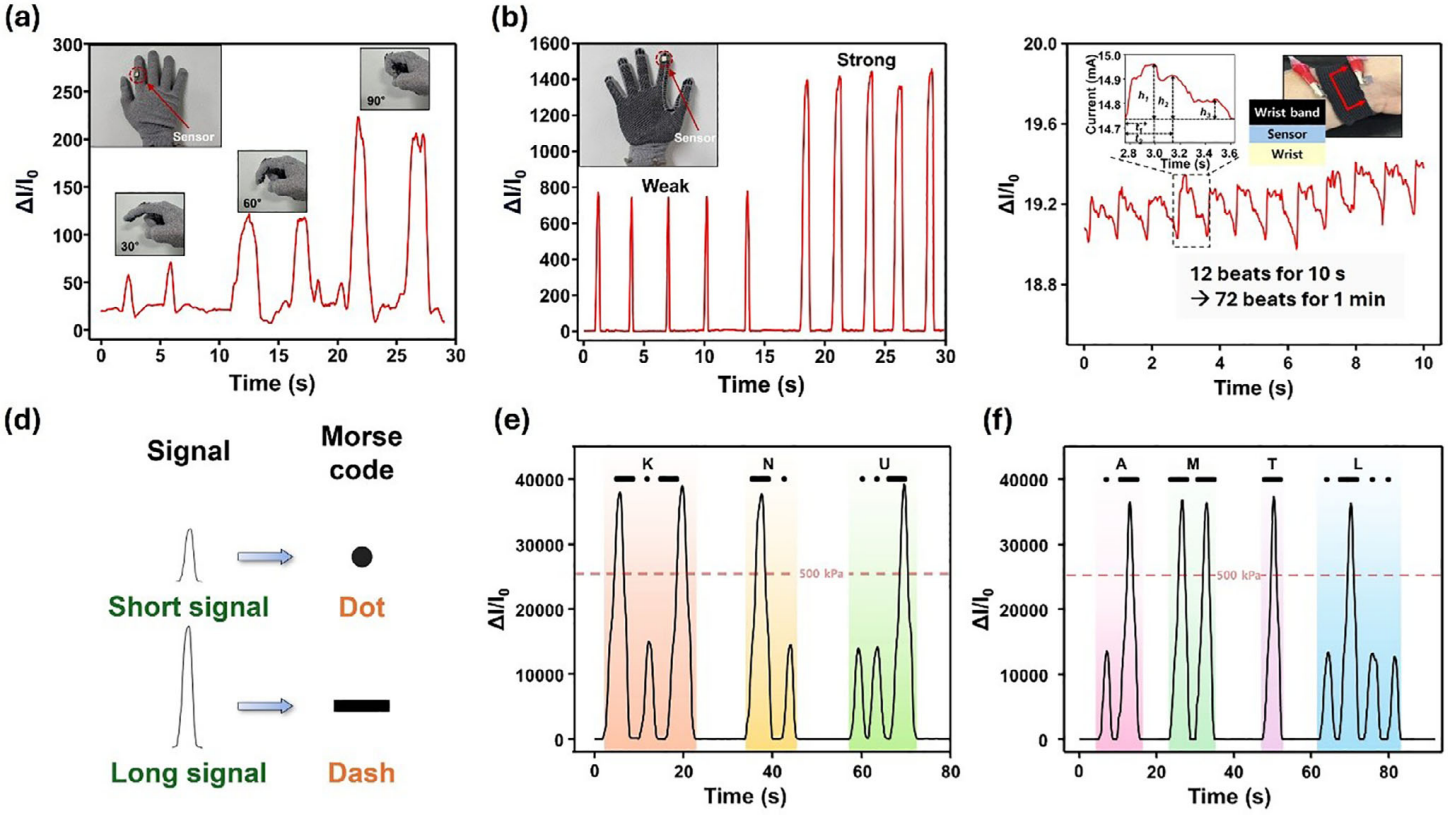
(a) Detection of finger joint movement using the sensor‐integrated glove. The sensor exhibited a proportional increase in relative current change with increasing joint bending angles of 30°, 60°, and 90°. (b) Relative current response of the fingertip sensor under weak and strong touch stimuli, showing stable and repeatable signals under repeated loading. (c) Real‐time detection of arterial pulse at the subject's wrist using the sensor‐integrated glove. Morse code demonstration based on pressure magnitude: (d) low and high pressures corresponding to dots and dashes, respectively, with the resulting output for (e) “KNU” and (f) “AMTL.” The red dashed line denotes the relative current change corresponding to an applied pressure of 500 kPa. The wide and sensitive pressure detection range enabled accurate and distinct classification of the input signals.

One advantage of our sensor is the use of mulberry paper and fabric as the sensing elements and electrodes. Since mulberry paper is commonly used in clothing and the sensor surface is covered with fabric, it can be easily integrated into wearable applications [71]. To demonstrate this, we integrated the sensor into a glove and a wristband for detecting human motion and physiological signals. Figure 4a,b presents the sensor‐integrated glove. When the finger was bent, the sensor experienced strain‐induced pressure. As shown in Figure 4a, the sensor attached to the finger joint effectively detected motion, exhibiting corresponding variations in current in response to applied strain. As the bending angles increased to 30°, 60°, and 90°, the sensor displayed a proportional increase in relative current change. Figure 4b shows the relative current change when the fingertip sensor was subjected to relatively weak and strong touches, with the sensor demonstrating stable and repeatable responses under both conditions. Figure 4c illustrates the real‐time response of the wrist‐worn sensor, showing an arterial pulse rate of approximately 72 beats per minute, consistent with that of a typical 24‐year‐old individual. The magnified pulse signal, detected at the subject's wrist, is shown in the inset of Figure 4c. To provide further insight into the physiological information contained in the measured pulse waveform, we analyzed the signal shown in Figure S20. The waveform exhibits three characteristic features commonly reported in digital volume pulse signals: the percussion (P), tidal (T), and diastolic (D) peaks. Based on these features, representative parameters related to arterial stiffness, namely, the radial augmentation index (AI_r_ = h_2_/h_1_), diastolic augmentation index (DAI_r_ = h_3_/h_1_), and digital volume pulse time (Δ*T*_DVP_ = t_2_ – t_1_), were calculated following previously reported definitions [72, 73]. The extracted values of AI_r_, DAI_r_, and Δ*T*_DVP_ were 0.767, 0.357, and 0.177 s, respectively. This analysis demonstrates that the proposed sensor can reliably acquire pulse waveforms and enable quantitative extraction of physiologically relevant parameters, serving as a proof‐of‐concept for wearable cardiovascular monitoring. Another advantage of the proposed sensor is its ability to detect a wide pressure range with high sensitivity. Unlike previous studies that demonstrated Morse code using either two separate sensors [21, 74] or by differentiating pressing duration at a fixed pressure level [75, 76], we distinguished dots and dashes solely based on the magnitude of applied pressure using a single sensor. This capability is enabled by the sensor's broad, non‐saturating pressure detection range, which allows reliable discrimination of distinct pressure amplitudes within a single sensing element. Accordingly, output signals were classified into lower and higher pressure regimes and mapped to the dots and dashes of Morse code, respectively, as shown in Figure 4d. A predefined pressure threshold of 500 kPa was used for classification: pressure inputs lower than 500 kPa were assigned as dots, whereas inputs equal to or higher than 500 kPa were assigned as dashes. Figure 4e,f presents the Morse code signals “KNU” and “AMTL,” respectively. To further evaluate repeatability, numerical Morse code patterns for digits 0–9 were generated 3 times each, resulting in 30 total trials (Figure S21). Under the tested conditions, all patterns were correctly classified according to the predefined pressure criterion, corresponding to a success rate of 100%. These results demonstrate the feasibility of pressure‐level‐based Morse code generation using a single tactile sensor.

**FIGURE 5:**
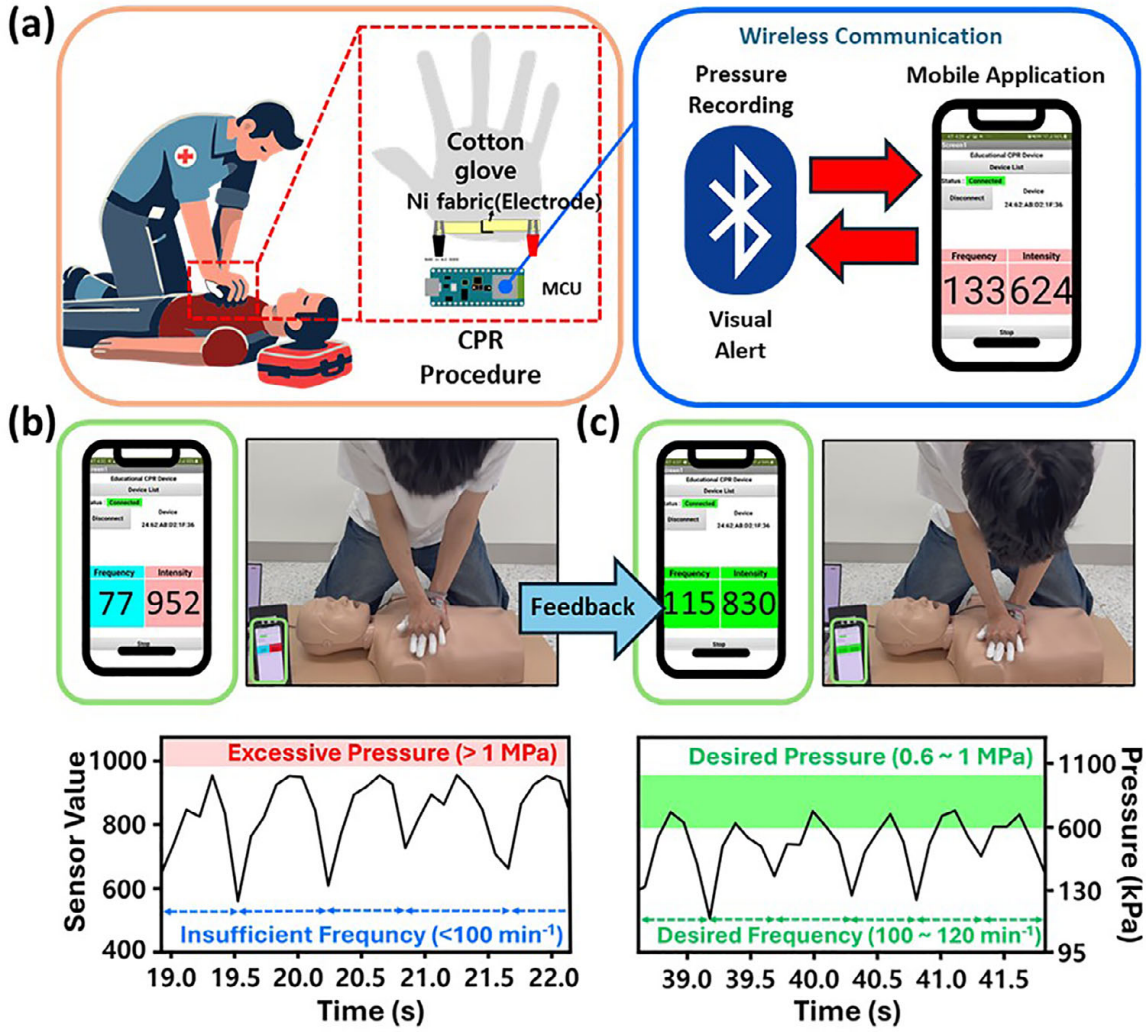
Proof‐of‐concept CPR training system based on high‐pressure detection capability. (a) Schematic of the proposed system comprising a sensor‐integrated cotton glove, microcontroller unit, and mobile application. Pressure applied to the palm is converted into a sensor signal corresponding to a specific pressure level and transmitted to the mobile application via Bluetooth communication. (b) Scenario demonstrating excessive chest compression pressure combined with insufficient frequency during CPR. (c) Scenario showing appropriate chest compression pressure and target frequency during CPR, achieved through real‐time feedback.

Cardiopulmonary resuscitation (CPR) is a critical emergency procedure performed to save individuals experiencing sudden cardiac or respiratory arrest. Delivering high‐quality chest compressions during CPR maintains the circulation of oxygenated blood to the brain and heart, thereby supporting the restoration of spontaneous heartbeat and improving the likelihood of patient recovery [77]. During CPR, chest compressions should be performed at a rate of 100–120 min^−1^ [78]. The corresponding pressure applied to the chest ranges from approximately 613 to 981 kPa, assuming a required force of 50 kgf over a palm contact area of 5 to 8 cm^2^ [79]. To this end, we developed a proof‐of‐concept CPR training system that leverages the sensor's wide pressure detection capability. Figure 5a provides an overview of the system, which comprises a sensor‐integrated cotton glove, a microcontroller unit, and mobile applications. The pressure applied to the palm is recorded as a sensor value corresponding to a specific pressure and transmitted to a mobile application via a Bluetooth communication module (see Movie S1 for a demonstration of the CPR training system). For instance, the person performing CPR exerted excessive pressure on the chest with an insufficient frequency, as shown in Figure 5b. An observer can then instruct the performer to adjust the compression frequency and pressure based on the visual alerts provided by the mobile application. Through this feedback process, the performer applies appropriate pressure at the desired frequency (Figure 5c). Based on these proof‐of‐concept demonstrations, our tactile sensor using a stacked structure of MXene‐coated mulberry paper presents a promising solution for wearable applications requiring a wide pressure detection range with high sensitivity.

## 3 Conclusion

We developed a flexible tactile sensor based on a stacked structure of mulberry paper coated with MXene. The hydrophilic nature of mulberry paper enabled the easy formation of electrically conducting MXene layers on the porous fiber network via simple dip‐coating. In addition, the rough surface and porous structure of the mulberry paper fibers increased the change in contact area compared to other paper substrates. The effects of the mulberry paper substrate, the number of stacked layers, and the amount of MXene on the fibers were characterized to evaluate pressure sensing performance. The sensor's performance, including response to various pressures, pressure application rates, response/relaxation times, long‐term stability, repeatability, and reproducibility, was evaluated under diverse loading conditions. Notably, our sensor exhibited high sensitivity exceeding 15 kPa^−1^ over a wide pressure range up to 1000 kPa, demonstrating a balanced combination of sensitivity and sensing range that remains challenging for previously reported MXene‐based paper tactile sensors. Owing to its high sensitivity, broad detection range, and fabric/paper‐based composition, we applied the sensor to wearable gloves and wristbands. Finally, integration into a CPR training system and a Morse code demonstration highlighted the advantages of our approach for wide‐range pressure detection. Given its high sensitivity, broad detection range, flexibility, simple fabrication method, and durability, the proposed sensor represents a promising solution for paper‐based electronics.

## 4 Experimental Section

### 4.1 MXene Preparation and Sensor Fabrication

All chemicals were used as received without further purification. Lithium fluoride (LiF) and hydrochloric acid (HCl, 37%) were purchased from Sigma–Aldrich Co., Ltd., and Ti_3_AlC_2_ MAX phase (99% purity, ∼325 mesh) was obtained from Uni Nano Tech Co., Ltd. Ti_3_C_2_T_x_ MXene was synthesized via the minimally intensive layer delamination (MILD) etching method. Initially, 1 g of LiF was dissolved in 20 mL of 9 m HCl, and the solution was stirred for at least 30 min to ensure complete dissolution. Subsequently, 1 g of Ti_3_AlC_2_ was gradually added to the etching solution over a 30 min period to prevent overheating. The resulting mixture was maintained at 35°C in an oil bath under continuous stirring (300 rpm) for 24 h to selectively remove the Al layers from the MAX phase. After etching, the suspension was washed with deionized water and centrifuged at 3500 rpm for 5 min. After each wash, the acidic supernatant was discarded, fresh deionized water was added, and the suspension was centrifuged again. This washing process was repeated until the supernatant reached a pH above 6. Upon neutralization, the black supernatant was collected by centrifugation at 3500 rpm for 20 min. The sediment, consisting of unetched Ti_3_AlC_2_ and multilayered Ti_3_C_2_T_x_, was discarded. The collected supernatant contained highly pure single‐ or few‐layered MXene sheets. The concentration of this supernatant was measured and subsequently used to fabricate the MXene‐coated mulberry paper for sensor integration.

To fabricate the MXene‐coated mulberry paper sensor, pre‐cut mulberry paper was dip‐coated (Ni‐Lo X2, Ni‐Lo Scientific) into the prepared MXene solution (5 mg/mL in DI water) at a withdrawal speed of 10 mm/s for 5 min. The coated paper was then dried in a vacuum oven (SH‐VDO‐08NG, SH‐Scientific) at 60°C for 1 h. After drying, the paper was cut into 10 × 10 mm^2^ squares and stacked using commercially available double‐sided tape. Commercial Ni‐coated fabric was used as the electrode material.

### 4.2 Characterization Methods

The structure and elemental composition of mulberry paper with and without MXene were examined using a scanning electron microscope (Regulus 8220, HITACHI). X‐ray diffraction (XRD) measurements were conducted on both uncoated and MXene‐coated mulberry paper using a Malvern Panalytical EMPYREAN diffractometer with Cu Kα radiation (λ = 1.5406 Å) at 40 kV and 30 mA. The thickness of the mulberry paper was measured using a digital micrometer (293‐821‐30, Mitutoyo), while surface roughness was evaluated using a confocal laser scanning microscope (LSM‐700, Carl Zeiss). To assess the pressure‐sensing performance, a push‐pull stand (ASM‐1000, Digitech) equipped with a force gauge (DTG‐10, Digitech) was employed to apply and measure force, and the corresponding current was recorded using a sourcemeter (2612B, Keithley) at an applied voltage of 1 V.

### 4.3 Wearable Application and CPR Training System

The pulse was measured by connecting a commercially available wristband to the Ni‐fabric electrodes of the sensor using silver threads, and recording the signal with a sourcemeter. To detect the pressure and motion of fingertips and finger joints, the sensor was integrated into a commercially available glove using silver threads, and measurements were also performed using the sourcemeter. A commercially available cotton glove was used to fabricate the CPR measurement glove, with the sensor placed and integrated on the palm side of the glove. For the CPR Training System, a microcontroller unit (MCU, Arduino Nano) with a Bluetooth module was attached to the wrist of the glove using Velcro. The MCU and the sensor electrodes were connected via electrical wires. The mobile application used in the CPR Training System was developed using MIT App Inventor. Written informed consent was obtained from all participants involved in the wearable device tests. All testing procedures conformed to the ethical requirements of Kyungpook National University.

## Conflicts of Interest

The authors declare no conflicts of interest.

## Supporting information




**Supporting File 1**: smll74205‐sup‐0001‐SuppMat.docx.

**Supporting File 2**: smll74205‐sup‐0002‐VideoS1.mp4.

## Data Availability

The data presented in the paper will be made available upon request to the corresponding author.
