# Supplementary material for: MXene‐Coated, Multi‐Layered Mulberry Paper‐Based Flexible Tactile Sensor With High Sensitivity Over a Wide Pressure Range
Source: Small. 2026 Jun 15;22(46):e74205. doi: 10.1002/smll.74205 (PMC13484976; doi:10.1002/smll.74205)
Supplement: Supplementary file 1 [file SMLL-22-e74205-s001.docx]

Supporting Information

MXene-Coated, Multi-Layered Mulberry Paper-Based Flexible Tactile Sensor with High Sensitivity over a Wide Pressure Range

Sangrim Lee, Chaemin Won, Jaebeen Ahn, Wonkeun Park, Eunsoo Ha, Jongbaeg Kim*, Taewook Kim*, Changyong Yim*, and Yunsung Kang*

**Contents**

1. Schematic of Ti_3_C_2_T_x_ MXene synthesis via the MILD method and fabrication process of the stacked MXene/mulberry paper sensor (Figure S1)
2. SEM image and EDX elemental mapping of mulberry paper (Figure S2)
3. Comparison of available product information and measured structural parameters of Type A, B, and C mulberry paper substrates. (Table S1)
4. SEM images of Type A, B, and C mulberry papers (Figure S3)
5. Pressure-sensing setup for the proposed tactile sensors (Figure S4)
6. Effect of MXene loading on pressure-sensing performance of Type B and C mulberry paper-based tactile sensors (Figure S5)
7. Relative current change of sensors composed of Kent and oil papers under applied pressure (Figure S6)
8. Cross-sectional SEM images of the three-layered MXene-coated mulberry paper sensor under different compression states. (Figure S7)
9. Relative current change of sensors with three-, four-, and five-layered structures under applied pressure (Figure S8)
10. Current response of the proposed sensor under applied pressures of (a) 98, 490, and 981 Pa using calibrated weights and (b) 1.962 Pa using a grain of rice (Figure S9)
11. Comparison of sensor performance in terms of sensitivity and sensing range with previously reported paper-based tactile sensors using MXene (Table S2)
12. Comparison of sensor performance in terms of sensitivity and sensing range with recently reported MXene-based tactile sensors (Figure S10)
13. Dynamic response characteristics under high-pressure conditions at 250 kPa and 500 kPa. (Figure S11)
14. Relative current change of the sensor under 2,500 repeated loading cycles (Figure S12)
15. Relative current change of sensors before and after harsh bending (1,000 cycles at ~90°) (Figure S13)
16. Relative current change of the sensor after 0, 500, and 1,000cycles of moderate bending with a bending radius of 0.6 cm (Figure S14)
17. Relative current change of the sensor under various temperature exposures (Figure S15)
18. Relative current change of the sensor under varying relative humidity conditions (Figure S16)
19. Functional reversibility of the sensor after exposure to extreme environmental conditions (Figure S17)
20. Reproducibility of the pressure-sensing performance of ten independently fabricated sensors (n = 10) (Figure S18)
21. EDX elemental mapping at three distinct positions of MXene-coated mulberry paper (Figure S19)
22. Real-time wrist pulse signal showing percussion (P), tidal (T), and diastolic (D) waves (Figure S20)
23. Repeatability evaluation of Morse code signal generation using numerical patterns (0–9) (Figure S21)

**Figure S1**. (a) Preparation of Ti_3_C_2_T_x_ MXene using a minimally intensive layer delamination method. A few-layered Ti_3_C_2_T_x_ MXene was obtained and dispersed in DI water. (b) Fabrication process of the proposed sensor. Mulberry paper was dip-coated with the Ti_3_C_2_T_x_ solution, vacuum-dried, cut into three pieces, and subsequently stacked and adhered using double-sided tape.

**Figure S2**. SEM image and EDX elemental mapping of mulberry paper in the absence of Ti_3_C_2_T_x_ MXene.

**Table S1.** Comparison of available product information and measured structural parameters of Type A, B, and C mulberry paper substrates.

| Sample | Type A | Type B | Type C |
| --- | --- | --- | --- |
| Source / product information | Commercial mulberry paper, product code: Kkonnipji | Commercial mulberry paper, product code: Unyongji | Commercial mulberry paper, product code: Okdangji |
| Manufacturing information | Proprietary / not disclosed | | |
| Surface roughness, Ra (µm) [N=3] | 23.261 ± 1.14 | 16.315 ± 0.7 | 10.915 ± 0.932 |
| Paper Thickness (µm) [N=3] | 300 ± 39.30 | 80 ± 3.06 | 123 ± 9.29 |
| Fiber Thickness (µm) [N=50] | 30.4 ± 15.1 | 20.9 ± 5.3 | 20 ± 5 |
| Basis weight (g m^−2^) [N=8] | 61.95 ± 3.15 | 27.7 ± 1.2 | 51.75 ± 2.5 |
| Apparent density (g cm^−3^) [N=8] | 0.233 ± 0.027 | 0.308 ± 0.07 | 0.413 ± 0.034 |
| SEM-based morphology | Rough, thick, porous fibrous network | Moderately compact fibrous network | Dense and relatively smooth fibrous network |

**Figure S3**. SEM images of mulberry paper types A, B, and C.

**Figure S4**. Experimental setup for evaluating the pressure-sensing performance of the proposed tactile sensors.

**Figure S5**. Effect of MXene loading on the pressure sensing performance of B-type (left) and C-type (right) mulberry paper-based tactile sensors.

**Figure S6**. Relative current responses of sensors fabricated using Kent paper and oil paper under varying applied pressures.

**Figure S7.** Cross-sectional SEM images of the three-layered MXene-coated mulberry paper sensor under different compression states. (a) Uncompressed, (b) moderately compressed, and (c) highly compressed states. The images qualitatively show progressive structural deformation from interlayer contact formation to intralayer fiber–fiber densification under compression.

**Figure S8**. Relative current change of sensors with three-, four-, and five-layered structures under applied pressure.

**Figure S9**. Current response of the proposed sensor under low-pressure loading: (a) stepwise application of 98, 490, and 981 Pa using calibrated load weights, and (b) ultra-low pressure of approximately 1.962 Pa applied using a grain of rice.

**Table S2**. Comparison of the sensitivity and sensing range of the developed sensor with previously reported paper-based tactile sensors incorporating MXene.

| Sensing materials | Substrate | Sensitivity (kPa^−1^) | Sensing range (kPa) | REF |
| --- | --- | --- | --- | --- |
| Ti_3_C_2_T_x_ | bacterial cellulose | 51.14 and 2.62 | 0–0.82 and 0.82–10.92 | [10] |
| Pt-infiltrated Ti_3_C_2_T_x_ | filter paper | 0.5 and 0.005 | 0–9 and 9–30 | [11] |
| Ti_3_C_2_T_x_ | bacterial cellulose | 81.7 and 12.5 | 0.001–0.01 and 0.01–10 | [16] |
| Ti_3_C_2_T_x_ | tissue paper | 0.55, 3.81, and 2.52 | 0.023–0.982, 0.982–10, and 10–30 | [17] |
| Ti_3_C_2_T_x_  /MoS_2x_Se_2(1−x)_ | cellulose paper | 13.043 | 1.477–3.185 | [18] |
| Ti_3_C_2_T_x_ | tissue paper | 509.5, 179.4, and 53.7 | 0.5–10, 10–30, and 30–100 | [19] |
| SnS  /Ti_3_C_2_T_x_ | cellulose paper | 7.49 | 1.5–3.2 | [20] |
| TeNWs  /Ti_3_C_2_T_x_ | cellulose paper | 9.29 | 1.5–3.2 | [21] |
| Ti_3_C_2_T_x_ | printing paper | 0.011 | 100–1100 | [22] |
| MoSe_2_  /Ti_3_C_2_T_x_ | cellulose paper | 14.7 | 1.477–3.185 | [23] |
| Graphene/Ti_3_C_2_T_x_/  sodium carboxymethyl cellulose | cellulose paper | 0.0162, 0.002352, and 0.000495 | 0–20, 20–130, and 130–270 | [24] |
| Ti_3_C_2_T_x_ | filter paper | 2.45, 0.83, and 0.19 | 0–20, 20–90, and 90–640 | [25] |
| Ti_3_C_2_T_x_ | mulberry paper | 245.4, 103.7, 46.3 and 17.9 | 1–10, 10–150, 150–350, and 350–1000 kPa | This study |

**Figure S10**. Comparison of the sensor’s sensitivity and sensing range with those of recently reported MXene-based tactile sensors.

**Figure S11.** Dynamic response characteristics under high-pressure conditions. Relative current change over time under applied pressures of 250 kPa and 500 kPa, respectively.

**Figure S12**. Relative current change of the sensor under 2,500 repeated loading–unloading cycles at 500 kPa.

**Figure S13**. Relative current change of the proposed sensor measured before and after 1,000 cycles of harsh bending at an angle of approximately 90°.

**Figure S14**. Relative current change of the sensor measured after 0, 500, and 1000 cycles of moderate bending with a bending radius of 0.6 cm.

**Figure S15**. Relative change in the current of the sensor under exposure to different temperatures.

**Figure S16**. Relative change in sensor current under exposure to various RH levels.

**Figure S17.** Functional reversibility of the sensor after exposure to extreme environmental conditions. Performance was evaluated after 30 minutes of exposure to (a) high temperature (60 °C), (b) high humidity (80% RH), and (c) combined high temperature and humidity (60 °C, 80% RH), followed by a return to ambient conditions. The consistency between the initial and post-exposure *ΔI/I_0_-*pressure curves demonstrates the sensor's excellent reversibility and reliability without the need for recalibration.

**Figure S18**. Reproducibility of the pressure-sensing performance of ten independently fabricated sensors (n = 10) prepared under the optimized conditions of Type A mulberry paper, 5 mg/mL MXene concentration, and a three-layered structure. Individual pressure-response curves and average sensitivity values with standard deviations for each pressure range are provided to evaluate device-to-device variation.

**Figure S19**. EDX elemental mapping at three distinct positions on the MXene-coated mulberry paper.

**Figure S20**. Real-time wrist pulse signal exhibiting characteristic features of the digital volume pulse waveform, including the percussion (P), tidal (T), and diastolic (D) peaks.

**Figure S21.** Repeatability evaluation of Morse code signal generation using numerical patterns (0–9). Each digit was generated 3 times based on the predefined pressure threshold (dot < 500 kPa, dash ≥ 500 kPa), resulting in a total of 30 trials. All numerical patterns were correctly classified with a success rate of 100%, demonstrating the high reliability and accuracy of the sensor for multi-level pressure signal categorization.
